# CD200 Positive Human Mesenchymal Stem Cells Suppress TNF-Alpha Secretion from CD200 Receptor Positive Macrophage-Like Cells

**DOI:** 10.1371/journal.pone.0031671

**Published:** 2012-02-20

**Authors:** Mika Pietilä, Siri Lehtonen, Elina Tuovinen, Kaarina Lähteenmäki, Saara Laitinen, Hannu-Ville Leskelä, Antti Nätynki, Juha Pesälä, Katrina Nordström, Petri Lehenkari

**Affiliations:** 1 Institute of Biomedicine, Department of Anatomy and Cell Biology, University of Oulu, Oulu, Finland; 2 Division of Surgery, Institute of Clinical Medicine, Department of Surgery and Intensive Care, University of Oulu and Clinical Research Centre, Oulu University Hospital, Oulu, Finland; 3 Finnish Red Cross Blood Service, Helsinki, Finland; 4 Aalto School of Chemical Technology, Department of Biotechnology and Chemical Technology, Espoo, Finland; Hemocentro de Ribeirão Preto, HC-FMRP-USP., Brazil

## Abstract

Human mesenchymal stem cells (hMSCs) display immunosuppressive properties *in vitro* and the potential has also been transferred successfully to clinical trials for treatment of autoimmune diseases. OX-2 (CD200), a member of the immunoglobulin superfamily, is widely expressed in several tissues and has recently been found from hMSCs. The CD200 receptor (CD200R) occurs only in myeloid-lineage cells. The CD200-CD200R is involved in down-regulation of several immune cells, especially macrophages. The present study on 20 hMSC lines shows that the CD200 expression pattern varied from high (CD200Hi) to medium (CD200Me) and low (CD200Lo) in bone marrow-derived mesenchymal stem cell (BMMSC) lines, whereas umbilical cord blood derived mesenchymal stem cells (UCBMSCs) were constantly negative for CD200. The role of the CD200-CD200R axis in BMMSCs mediated immunosuppression was studied using THP-1 human macrophages. Interestingly, hMSCs showed greater inhibition of TNF-α secretion in co-cultures with IFN-γ primed THP-1 macrophages when compared to LPS activated cells. The ability of CD200Hi BMMSCs to suppress TNF-α secretion from IFN-γ stimulated THP-1 macrophages was significantly greater when compared to CD200Lo whereas UCBMSCs did not significantly reduce TNF-α secretion. The interference of CD200 binding to the CD200R by anti-CD200 antibody weakened the capability of BMMSCs to inhibit TNF-α secretion from IFN-γ activated THP-1 macrophages. This study clearly demonstrated that the efficiency of BMMSCs to suppress TNF-α secretion of THP-1 macrophages was dependent on the type of stimulus. Moreover, the CD200-CD200r axis could have a previously unidentified role in the BMMSC mediated immunosuppression.

## Introduction

The immune system can be classified into adaptive and innate immune responses and their complex interplay is crucial to achieve properly working defense system [1], [2]. Macrophages are central players in innate immunity and they are classically divided into the two phenotypes M1 (proinflammatory) and M2 (healing) macrophages [1]–[3]. However, recent studies have shown that the heterogeneity of macrophages is much wider and complex than has previously been thought [4]. Bacterial lipopolysaccharide (LPS) is commonly used to induce the M1 phenotype and further the secretion of T helper 1 (Th1) cytokines, such as tumor necrosis factor-alpha (TNF-α), interleukin-1β or interleukin-6 [3]. Interferon-gamma (IFN-γ) is also an important activator or primer of macrophages and other immune cells [5]. IFN-γ is mainly produced by Th1-cells and natural killer cells. The presence of IFN-γ has been shown to be elevated in many inflammatory conditions and also to be relevant during macrophage activation in several autoimmune diseases [3], [6]–[9]. A recent proteome bioprofiling study has also revealed differences between LPS and IFN-γ activation of macrophages, in which the un-stimulated macrophages could be distinguished from IFN-γ primed and LPS activated ones [10].

Human mesenchymal stem cells (hMSCs) have shown immunosuppressive properties which are mediated through modifying both innate and adaptive immune systems [11]–[15]. hMSC-based cellular therapies have shown their usefulness in treatment of autoimmune diseases, such as Crohn's disease, graft versus host disease (GVHD) and diabetes [16]–[19]. The role of different soluble factors secreted by hMSCs, such as kynurenines produced by the tryptophan-degrading enzyme indoleamine-2,3-dioxygenase-1 (IDO1), prostaglandin E2 (PGE2), transforming growth factor beta (TGF-β) and galectin-1 have been demonstrated [11], [20]–[22].

Recently the cell-cell interactions between hMSCs and different immune cells have also been studied and the role of the intercellular adhesion molecule-1 (ICAM-1), the vascular cell adhesion molecule-1 (VCAM-1) and Thy-1 (CD90) has been shown in hMSC mediated immunosuppression [23], [24]. OX-2 (CD200), a membrane glycoprotein which belongs to the immunoglobulin superfamily, shows a broad expression pattern and recently it has been suggested to be a marker of native hMSC population [25]–[27]. On the other hand, expression of the CD200 receptor (CD200R) is restricted only to the myeloid lineage cells [28] and CD200 binding to the CD200R is shown to suppress the activity of many immune cells but especially macrophages [29]–[32]. Murine knock-out models have demonstrated the importance of the CD200-CD200R axis in controlling macrophage activity also *in vivo* and the immunosuppressive capacity of CD200R agonist has been demonstrated *in vitro* and also *in vivo* in collagen induced arthritis models [29], [33]–[35]. In addition, murine knock-out models in skin graft experiments have shown the important role of the CD200 interaction with CD200R in skin engraftment [36].

Only a few studies have previously examined the interactions between human macrophages and hMSCs [37], [38], whereas the CD200-CD200r axis has been shown to be relevant especially in regulation of macrophages. Accordingly, the present study strives to elucidate the role of the CD200-CD200R axis in bone marrow-derived mesenchymal stem cells (BMMSCs) mediated immune modulation of THP-1 macrophage-like cells.

## Methods

### hMSC Isolation and Culture

Northern Ostrobothnia Hospital District Ethical committee has approved the collection of human mesenchymal stem cells from patients from Oulu University Hospital after written consent. Isolation and culture of BMMSCs: BMMSCs were isolated from an unaffected bone site of patients who were operated for osteoarthritis and from some younger patients operated for idiopathic scoliosis as described earlier [39]. To control the quality of BMMSC lines, patients with neuromuscular scoliosis, autoimmune diseases such as rheumatoid arthritis, or genetic diseases were excluded. Samples from bone marrow were suspended in a proliferation medium containing alpha minimum essential medium (αMEM) buffered with 20 mM HEPES and containing 10% heat-inactivated fetal bovine serum (FBS), 100 U/ml penicillin, 0.1 mg/ml streptomycin and 2 mM L-glutamine, and the suspension was transferred into cell culture flask. hMSCs were allowed to attach for 48 hours at 37°C under 5% CO_2_ and 20% O_2_. Nonattached cells were removed by changing fresh medium and attached cells were cultured in bottom of flask until they reached 70–80% confluence. The medium was changed twice a week and cells in passages 3–6 were used in experiments.

Cord blood collections were performed at the Helsinki University Central Hospital, Department of Obstetrics and Gynaecology, and Helsinki Maternity Hospital after written consent and study protocol was approved by ethical review board of Helsinki University Central Hospital and the Finnish Red Cross Blood Service. Isolation and culture of umbilical cord blood-derived mesenchymal stem cells (UCBMSCs): Cord blood collections were performed at the Helsinki University Central Hospital (HUCH), Department of Obstetrics and Gynaecology, and Helsinki Maternity Hospital. UCBMSCs were isolated and expanded as described earlier [40]. Mononuclear cells were isolated using Ficoll-Hypaque (Amersham Biosciences, Piscaway, NJ, USA) gradient centrifugation. Tissue culture plates (Nunc) were coated with fibronectin (Sigma) and mononuclear cells were plated at a density of 1×10^6^/cm2. The initial UCBMSC line establishment was performed under hypoxic conditions (5% CO_2_, 3% O_2_ at 37°C) in medium containing αMEM with Glutamax (Gibco, Grand Island, NY, USA) and 10% fetal calf serum (Gibco) supplemented with 10 ng/ml epidermal growth factor (EGF, Sigma), 10 ng/ml recombinant human platelet-derived growth factor (rhPDGF-BB; R&D Systems, Minneapolis, MN, USA), 50 nM Dexamethasone (Sigma), 100 U/ml penicillin together with 100 µ g/ml streptomycin (Invitrogen). Cells were allowed to adhere overnight and non-adherent cells were washed out with medium changes. Hence, UCBMSCs were cultured under normoxic conditions (5% CO_2_ and 20% O_2_ at 37°C) and proliferation media was renewed twice a week. Established lines were passaged when almost confluent and replated at 1000–3000 cells/cm^2^. Cells in passages 3–7 were used.

### Differentiation of THP-1 Monocytes into Macrophage-like Cells and Co-culture with hMSCs

The THP-1 human monocyte cell line from an acute monocytic leukemia patient was obtained from ATCC (TIB-202) and cells were expanded in RPMI 1640 medium (Lonza) supplemented with 10% heat-inactivated FBS, 100 U/ml penicillin, 0.1 mg/ml streptomycin and 2 mM L-glutamine (expansion medium). THP-1 monocytes were first cultured 2–3 weeks in T15 flask before their differentiation was started. Fresh expansion medium was added twice a week and at the end of the week cells were transferred to the new T15 flask. To start differentiation, THP-1 cells were suspended in the expansion medium supplemented with 100 ng/ml phorbol 12-myristate 13-acetate (PMA, Sigma Aldrich) and then 100 000 cells per well were added on 24-well plates and were incubated for 48 h at 37°C under 5% CO_2_ and 20% O_2_. After 48 hours, most of the cells were attached to the bottom of plates and PMA containing medium was removed. THP-1 macrophage like cells were washed twice with phosphate-buffered saline (PBS) followed by addition of co-culture assay medium containing αMEM buffered with 20 mM HEPES and supplemented with 100 U/ml penicillin, 0.1 mg/ml streptomycin and 2 mM L-glutamine. hMSCs were expanded as described above and detached from the culture flask and suspended in the co-culture assay medium. To start the co-culture, 100 000 hMSCs per well were added (the final ratio of hMSCs and THP-1 macrophage cells were 1∶1) in 24-well plate where PMA-treated THP-1 macrophage-like cells already were plated and after that 100 ng/ml LPS (purified from Escherichia coli 055:B5; Sigma Aldrich) or 100 ng/ml IFN-γ (Sigma Aldrich) were added. During co-culture, the total volume of medium in 24-well plate wells was 0.5 ml. Co-culture was performed for 24 hours at 37°C under 5% CO_2_ and 20% O_2_ followed by the collection of supernatants for cytokine assays. Controls included hMSCs alone, hMSCs with unstimulated THP-1 macrophages, hMSCs in presence of 100 ng/ml LPS or 100 ng/ml IFN-γ to see whether hMSCs also secrete TNF-α. In addition, unstimulated THP-1 macrophage-like cells, 100 ng/ml LPS activated THP-1 macrophage-like cells and 100 ng/ml IFN-γ activated THP-1 macrophage-like cells were included as controls in which the co-cultures were compared. All co-cultures and controls were performed in three replicates. The change in TNF-α or IL-10 secretion was determined by comparing cytokine levels in co-culture with hMSCs in the presence of LPS or IFN-γ to the cytokine levels in THP-1 macrophage-like cells alone in the presence of LPS or IFN-γ. Co-culture experiments were repeated 2–3 times and results are represented as a mean±SD of three independent replicates of one experiment unless otherwise indicated.

Mouse anti-human PE-conjugated CD200 antibody (BD Biosciences) was used in some experiments to block CD200 in hMSCs. For blocking, the hMSCs were incubated with anti-human CD200 antibody for 20 minutes in PBS with 0.5% bovine serum albumin (BSA) and then washed twice with PBS and suspended in co-culture assay medium whereas the control cells of the same hMSC pool remained untreated.

### Immunofluorescent Stainings

hMSCs were plated on sterile coverslips at a density of 5000 cells/well in 24-well plates in the proliferation medium and were grown for 2–3 days. The medium was removed and cells were fixed with 3% paraformaldehyde in PBS for 15 min followed by washing twice with PBS. The cells were first incubated in PBS with 1.0% BSA for 1 hour at room temperature to block unspecific binding. Phycoerythrin (PE) conjugated mouse anti-human CD200 and fluorescein isothiocyanate (FITC) conjugated mouse anti-human CD90 (Stem Cell Technologies Inc) were diluted in PBS with 1.0% BSA (1∶10) and cells were incubated over night at 4°C in dark and shaker. After incubation, the cells were washed three times with PBS for 5 minutes and then incubated with Hoechst in PBS with 1.0% BSA (1∶1000) for 5 minutes in dark. Hoechst was washed away with PBS and coverslips were mounted to glass slides.

THP-1 cells were seeded on sterile coverslips at 100 000 cells/well in 24-well plates and differentiated as stated above. The differentiation media was removed and cells were washed and incubated for 24 hours in assay medium alone, assay medium with 100 ng/ml LPS and assay medium with 100 ng/ml IFN-γ. After 24 hours, medium was removed and cells were fixed with 3% paraformaldehyde for 15 minutes and washed twice with PBS. The cells were first blocked in PBS with 1.0% BSA for 1 hour at room temperature. PE conjugated monoclonal anti-human CD200R antibody (eBioscience) was diluted in PBS with 1.0% BSA (1∶10) and cells were incubated overnight at 4°C with shaking in dark. Hoechst staining and mounting were performed as for hMSCs. THP-1 macrophage-like cells and hMSCs were photographed using an Axio Lab.A1 microscope (Carl Zeiss MicroImaging Gmbh, Germany).

### Flow Cytomeric Analysis of Cell Surface Antigens

hMSCs were detached from the culture flask and suspended in PBS with 0.5% BSA. The characterization of cell surface antigens for hMSCs was performed by using the following conjugated antibodies: CD44 (fluorescein isothiocyanate (FITC); BD Biosciences), CD49e (phycoerythrin (PE); BD Biosciences), CD90 (FITC; Stem Cell Technologies, Grenoble, France), CD73 (PE; BD Biosciences), HLA-ABC (allophycocyanin (APC; BD Biosciences), CD54 (APC; BD Biosciences) and CD105 (FITC; Abcam, Cambridge, United Kingdom). Negative surface antigens for hMSCs were incubated simultaneously as a group in the same sample: HLA-DR (PE; BD Biosciences), CD34 (PE; BD Biosciences), CD45 (PE; BD Biosciences), CD14 (PE; BD Biosciences), and CD19 (PE; BD Biosciences). Samples were analyzed by using FACSCalibur (Becton Dickinson), equipped with dual lasers emitting at 488 nm and 633 nm. The fluorescence emissions were measured at 585 nm±42 nm and data were analyzed with FlowJo (TreeStar Inc. Ashland, OR). The gates for positivity were individually defined by a negative control due to the differences in autofluorescence between different hMSC lines.

### Analysis of Cytokine Secretion by Enzyme-linked Immunosorbent Assay (ELISA)

Supernatants were collected after 24 hours from hMSCs and THP-1 cells co-cultures and stored at −70°C until analysis. TNF-α and IL-10 levels were determined from supernatants as described below. TNF-α and IL-10 capture antibodies (TNF-α, BD Pharmingen; IL-10, BD Pharmingen) were diluted to 1 µ g/ml and incubated in maxi-sorp 96-well plates (Nunc) over night at 4°C. Following incubation, unbound capture antibodies were removed and wells were blocked with PBS with 1.0% BSA (Sigma) for 1 hour. Plates were washed with PBS supplemented with Tween-20 (Sigma) and IL-10 and TNF-α standards (TNF-α, BD Pharmingen; IL-10, BD Pharmingen) together with collected supernatants followed by thawing and incubation in 96-well maxi-sorp plates for two hours at room temperature. Samples were also analyzed as 1∶10 dilutions. Standards and supernatants were diluted in the co-culture assay medium. After two hours of incubation, standards and samples were removed and plates were washed five times with PBS containing Tween-20. Biotin-labeled IL-10 and TNF-α antibodies (IL-10, BD Pharmingen; TNF-α, BD Pharmingen) were diluted to 0.25 µ g/ml in PBS with 1.0% BSA and Tween-20 and incubated for one hour at room temperature. Wells were washed five times with PBS+Tween-20. Avidin-horseradish peroxidase (HRP) conjugate (BD Pharmingen) was diluted in PBS with 1.0% BSA and Tween-20 (1∶1000) and was incubated for 30 minutes. Avidin-HRP was removed and wells were washed five times with PBS with Tween-20. Substrates of 3,3′, 5,5′ tetramethylbenzidine (TMB, PD Pharmingen) reagent set were mixed in 1∶1 and added to the wells and color development was followed at 1 hour for IL-10 and 15 minutes for TNF-α. The reaction was stopped with 1 M phosphoric acid and absorbance was measured at 450 nm with ELISA plate-reader (MultiScan). Cytokine concentrations were determined by MultiCalc software. Results from co-cultures are indicated as %-change in cytokine secretion when compared to activated THP-1 macrophage-like cells alone.

### Statistics

Statistical analysis and all diagrams were performed by using the OriginPro version 8. All data are presented as mean ± standard deviation (SD) of the results from three independent replicates or three different hMSC lines unless otherwise indicated. The significance level was determined by two-sample *t*-test and one way analysis of variance (ANOVA) when CD200 high, medium and low BMMSCs were compared together. The p-value p<0.05 was considered as statistically significant.

## Results

### CD200 Expression Pattern Differences in hMSC Lines

**Figure 1:**
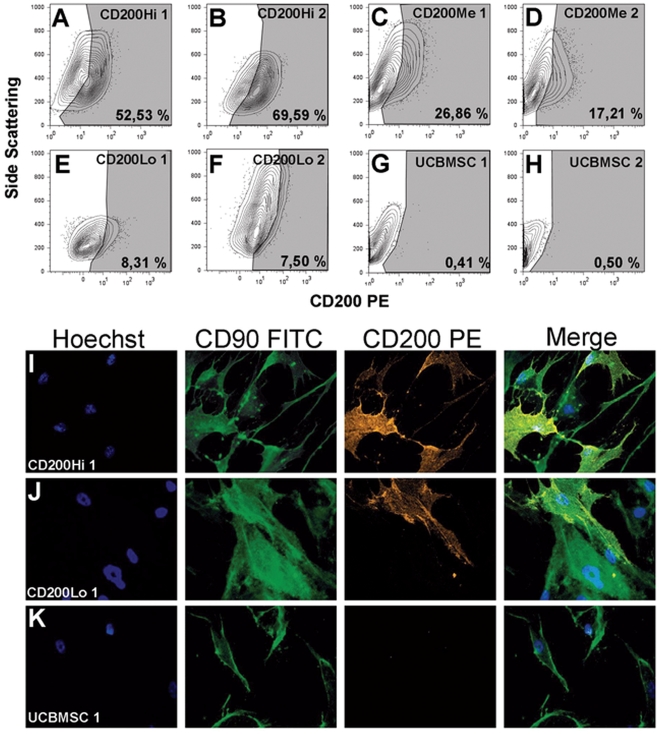
CD200 expression in hMSCs. Flow cytometry analysis showed that hMSC lines had different proportion of cells positive for CD200. **A**) CD200Hi 1 BMMSC, **B**) CD200Hi 2 BMMSC, **C**) CD200Me 1 BMMSC, **D**) CD200Me 2 BMMSC, **E**) CD200Lo 1 BMMSC, **F**) CD200Lo 2 BMMSC, **G**) UCBMSC 1 and **H**) UCBMSC 2. The CD200 positivity was determined by a negative control individually since the different autofluorescent of hMSC lines. An immunofluorescent staining of cell surface antigens showed that hMSCs labeled with PE-conjugated anti-human CD200 (red) and FITC-conjugated anti-human CD90 (green) express CD200 differentially whereas they were all positive for CD90, **I**) CD200Hi 1 BMMSC (magnification 400×), **J**) CD200Lo 1 BMMSC (magnification 400×) and **K**) UCBMSC 1 (magnification 400×). CD200-PE and CD90-FITC were stained against nuclear marker Hoechst (Blue).

BMMSCs differed in CD200 expression and high (CD200Hi), medium (CD200Me) or low (CD200Lo) positivity for CD200 could be identified (**Table 1**). On the other hand, the three studied UCBMSC lines were CD200 negative. Based on flow cytometry analysis of the 20 different hMSC lines, the role of the CD200-CD200R axis in hMSCs mediated immunosuppression was further elucidated with following hMSC lines: CD200Hi 1 (**Figure 1 A**; 52.53% positivity for CD200), CD200Hi 2 (**Figure 1 B**; 69.59% positivity for CD200), CD200Me 1 (**Figure 1 C**; 26.86% positivity for CD200), CD200Me 2 (**Figure 1 D**; 17.21% positivity for CD200), CD200Lo 1 (**Figure 1 E**; 8.31% positivity for CD200), CD200Lo 2 (**Figure 1 F**; 7.50% positivity for CD200), UCBMSC 1 (**Figure 1 G**; 0.41% positivity for CD200) and UCBMSC 2 (**Figure 1 H**; 0.50% positivity for CD200). hMSCs were positive for the cell surface antigens CD90, CD73, CD105, CD49e, CD44 and HLA-ABC, and negative for CD34, CD19, CD45, HLA-DR and CD14 (data not shown). All eight hMSC lines therefore conformed with the surface marker definition assembled by the International Society for Cellular Therapies [41]. The CD200 expression was also confirmed by immunofluorescent staining in which CD200Hi 1 (**Figure 1 I**), CD200Lo 1 (**Figure 1 J**) and UCBMSC 1 (**Figure 1 K**) were selected to demonstrate the differences in CD200 expression on cell surface. For CD200Hi 1, most of the cells were positive for both CD200 and CD90, whereas for CD200Lo 1 only a few cells were positive for CD200 and UCBMSC 1 were completely negative. Regardless, CD200Lo 1 and UCBMSC 1 were CD90 positive. The further passage of the cells *in vitro* did not change dramatically the CD200 expression on the cell surface of hMSCs as can be seen with the CD200Hi 2 BMMSC line (**Table 2**).

**Table 1:** Flow cytometry analysis of CD200 expression in hMSCs.

hMSC Lines	hMSC Lines Selected in Experiments	Positivity for CD200 (%)	Classification based on CD200	Source	Age (year) and Gender
hMSC 1	CD200Hi 1	52.53	CD200Hi	Bone Marrow	58, female
hMSC 2	CD200Hi 2	69.59	CD200Hi	Bone Marrow	16, female
hMSC 3	CD200Me 1	26.86	CD200Me	Bone Marrow	35, female
hMSC 4	CD200Me 2	17.21	CD200Me	Bone Marrow	16, male
hMSC 5	CD200Lo 1	7.50	CD200Lo	Bone Marrow	10, male
hMSC 6	CD200Lo 2	8.31	CD200Lo	Bone Marrow	70, male
hMSC 7	UCBMSC 1	0,50	UCBMSC	Umbilical Cord Blood	0, male
hMSC 8	UCBMSC 2	0.41	UCBMSC	Umbilical Cord Blood	0, male
hMSC 9	-	0.40	UCBMSC	Umbilical Cord Blood	0, female
hMSC 10	-	11.41	CD200Me	Bone Marrow	63, female
hMSC 11	-	26.45	CD200Me	Bone Marrow	20, male
hMSC 12	-	27.18	CD200Me	Bone Marrow	69, male
hMSC 13	-	11.64	CD200Me	Bone Marrow	54, male
hMSC 14	-	5.95	CD200Lo	Bone Marrow	55, male
hMSC 15	-	13.58	CD200Me	Bone Marrow	17, female
hMSC 16	-	16.17	CD200Me	Bone Marrow	40, male
hMSC 17	-	8.62	CD200Lo	Bone Marrow	62, female
hMSC 18	-	28.17	CD200Me	Bone Marrow	58, male
hMSC 19	-	11.95	CD200Me	Bone Marrow	17, female
hMSC 20	-	14.67	CD200Me	Bone Marrow	38, male

Positivity for CD200 was determined by flow cytometry and a negative control was used individually by each line in gating since the different autofluorescence of hMSC lines.

Abbreviations: hMSCs, human mesenchymal stem cell; CD200Hi, bone marrow derived mesenchymal stem cell line with 50–70% positivity for CD200; CD200Me, bone marrow derived mesenchymal stem cell line with 10–29% positivity for CD200; CD200Lo, bone marrow derived mesenchymal stem cell line with 2–9% positivity for CD200; UCBMSC, umbilical cord blood derived mesenchymal stem cells CD200 negative.

**Table 2:** Flow cytometry analysis of CD200 expression in different passages.

CD200Hi 2 BMMSC line	Positivity for CD200 (%)
Passage 1	57.7
Passage 2	69.6
Passage 4	67.8
Passage 6	68.0

Positivity for CD200 was determined by flow cytometry and a negative control was used in gating.

Abbreviations: CD200Hi 2 BMMSC Line, bone marrow derived mesenchymal stem cell line with 50–70% positivity for CD200.

### IL-10 and TNF-α Secretion from Macrophage-like Cells Is Modulated by CD200Hi 1 BMMSC Line

**Figure 2:**
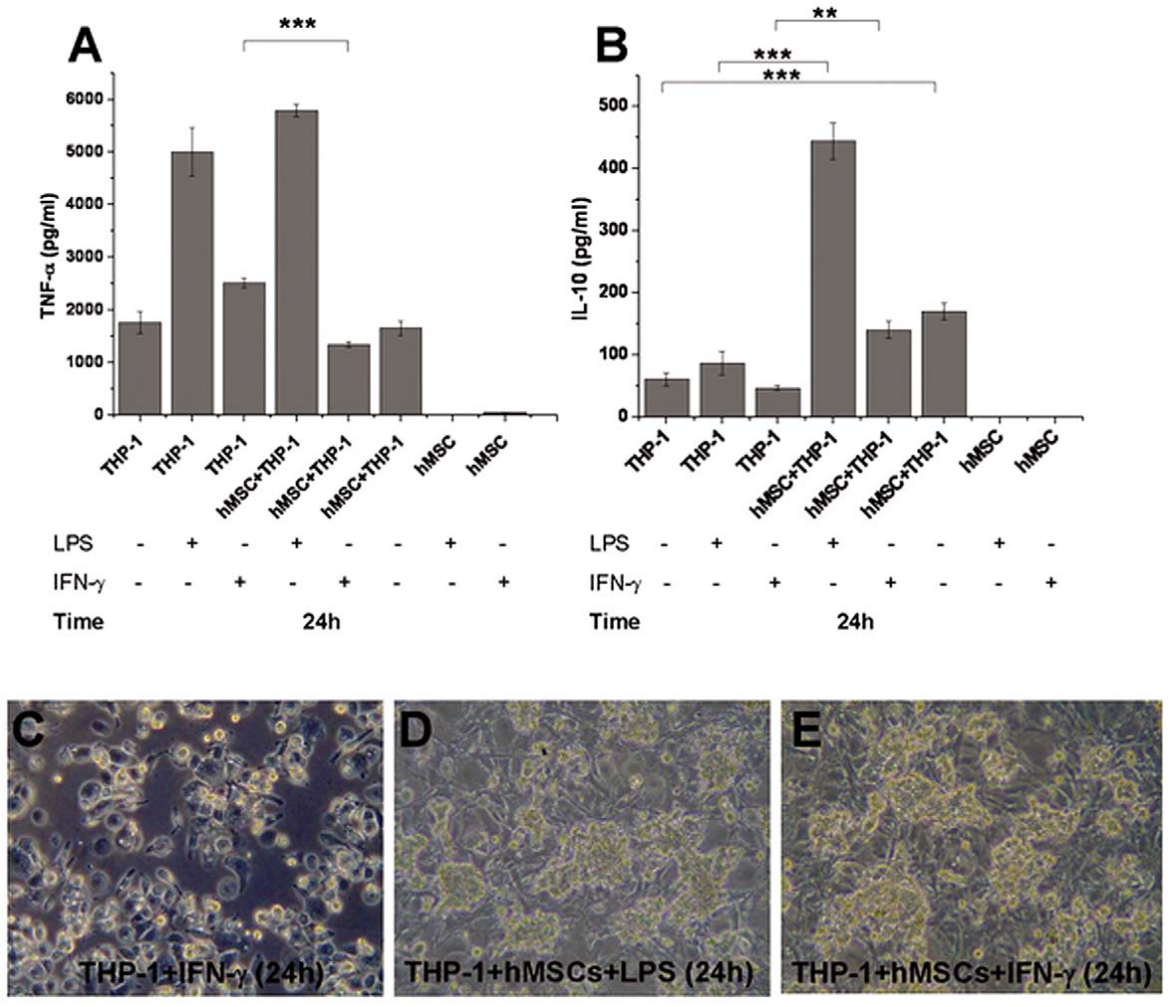
An exemplary co-culture experiment shows that hMSCs can modulate cytokine secretion of THP-1 macrophage-like cells. **A**) CD200Hi 1 were co-cultured with THP-1 macrophage-like cells in presence and absence of LPS (100 ng/ml) or INF-γ (100 ng/ml) and TNF-α secretion was measured. The co-culture of CD200Hi 1 with THP-1 macrophage-like cells decreased the TNF-α secretion when INF-γ was present whereas there were no significant differences in TNF-α secretion in the presence of LPS when compared to THP-1 macrophage-like cells alone. **B**) The co-culture of CD200Hi 1 with THP-1 macrophage-like cells significantly increased IL-10 secretion in all conditions when compared to THP-1 macrophage-like cells alone. hMSC lines did not secreted significant levels of IL-10 or TNF-α in any conditions. Results are represented as a mean±SD of three independent replicates. **C**) Light microscope analysis of THP-1 macrophage-like cells after 24 hours culture revealed that when THP-1 macrophage-like cells were cultured alone they were attached to the bottom of the wells and showed macrophage-like morphology (magnification 200×) where as in co-culture with hMSCs in presence of **D**) LPS or **E**) IFN-γ they seemed to form aggregates on the top of hMSCs monolayer (magnification 200×). Before co-culture, THP-1 macrophage-like cells were differentiated for 48 hours in PMA (100 ng/ml) supplemented medium as described in materials and methods. ** and *** indicates two-sample t-test p-values p<0.01 and p<0.001 respectively.

THP-1 cells were differentiated into the macrophage-like cells by incubating for 48 hours in PMA (100 ng/ml) containing medium. The differentiation was seen as an attachment of the cells to the bottom of culture plates and an induced secretion of TNF-α and IL-10 (**Figure 2** **.**). The addition of LPS increased TNF-α secretion (mean±SD of three independent replicates) after 24 h culture whereas IFN-γ caused only mild increase or had no effect on the TNF-α secretion when compared to un-stimulated THP-1 macrophage-like cells alone. The TNF-α concentration increased from 1763.5±207.2 pg/ml to 5002.3±455 pg/ml after addition of LPS and from 1763.5±207.2 to 2510.3±92.2 pg/ml after addition of IFN-γ respectively (**Figure 2A**). The co-culture with hMSCs inhibited TNF-α secretion from IFN-γ activated THP-1 macrophage-like cells from 2510.3±92.2 pg/ml to 1337.0±53.7 pg/ml (p<0.001) whereas hMSCs had no significant impact on TNF-α secretion of LPS activated THP-1 macrophage-like cells where TNF-α concentration was 5002.3±455 pg/ml in activated THP-1 cells alone and 5790.5±116.7 pg/ml in co-culture with hMSCs. LPS or IFN-γ did not induce significant IL-10 secretion from THP-1 macrophage-like cells (**Figure 2B**). However, the addition of hMSCs induced IL-10 secretion of THP-1 macrophage-like cells in the presence of both activators. IL-10 concentration changed from 86.9±18.6 pg/ml to 444.34±29.9 pg/ml in the presence of LPS (p<0.001) and from 47.0±3.8 pg/ml to 140.6±14.0 pg/ml (p<0.01) in the presence of IFN-γ (**Figure 2B**). The hMSC lines showed negligent IL-10 or TNF-α secretion under all tested condition. When THP-1 differentiated macrophage-like cells were grown alone their morphology resembled macrophages (**Figure 2C**). In co-cultures, hMSCs were always attached into the bottom of culture plates and most of the THP-1 macrophage-like cells seem to form aggregates on the top of the hMSCs (**Figure 2DE**).

### IFN-γ Activated THP-1 Macrophage-like Cells Are Sensitive to hMSC Mediated Inhibition of TNF-α Secretion

**Figure 3:**
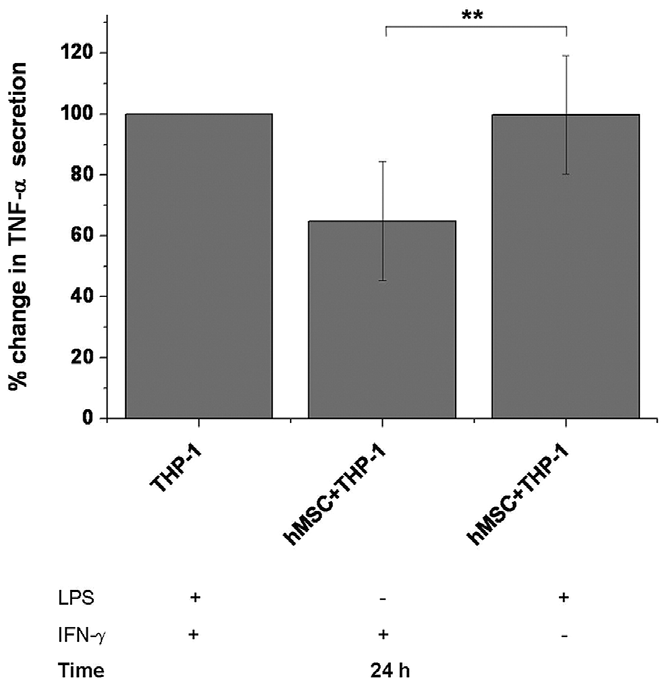
hMSCs inhibit TNF-α secretion from macrophage-like cells more efficiently in the presence of IFN-γ. When hMSCs were co-cultured with THP-1 macrophage-like cells for 24 hours in the presence of IFN-γ the decrease in TNF-α secretion was significantly greater when compared to co-culture in the presence of LPS. Results are represented as a mean±SD of co-cultures of eight different hMSC lines. The TNF-α concentration from supernatants of LPS and IFN-γ stimulated THP-1 macrophage-like cells alone were indicated as 100% and TNF-α concentration of supernatants from co-cultures with different hMSC lines were compared to that. Before experiment, THP-1 macrophage-like cells were differentiated 48 hours in PMA (100 ng/ml) supplemented medium as described in materials and methods. ** indicates two-sample t-test p-value p<0.01.

The ability of hMSCs to inhibit TNF-α secretion differed between LPS and IFN-γ stimulated THP-1 macrophage-like cells. The eight hMSC lines were co-cultured for 24 hours with THP-1 macrophage-like cells in the presence of LPS or IFN-γ and TNF-α secretion from THP-1 macrophage-like cells in co-cultures were compared to TNF-α secretion of IFN-γ or LPS activated THP-1 macrophage-like cells alone (**Figure 3**). The presence of hMSCs decreased TNF-α secretion from IFN-γ activated THP-1 macrophage-like cells from 100% to 64.9%±19.5% whereas when hMSCs were co-cultured with LPS activated THP-1 macrophage-like cells there were practically no differences in TNF-α secretion when compared to LPS activated THP-1 macrophage-like cells alone. The decrease in TNF-α secretion by presence of hMSCs was greater in IFN-γ primed THP-1 macrophage-like cells when compared to LPS activated ones (p<0.01).

### CD200 Expression is Linked to the Capability of BMMSCs to Inhibit TNF-α Secretion from IFN-γ Activated Macrophage-like Cells

**Figure 4:**
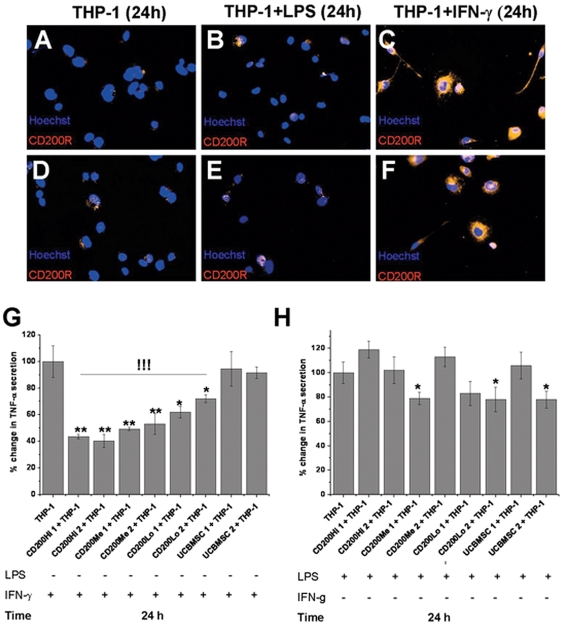
Immunnohistochemical analysis of CD200R expression in macrophages, the role of CD200-CD200R axis in hMSCs mediated immunosuppression. The immunofluorescent staining with PE-conjugated anti-human CD200R (red) against nuclear marker Hoechst (blue) revealed interesting differences in CD200R expression between **A,D**) un-stimulated (24 h culture without LPS or IFN-γ) THP-1 macrophage-like cells (magnification 400×) **B,E**) LPS stimulated (100 ng/ml for 24 h) THP-1 macrophage-like cells (magnification 400×) and **C,F**) IFN-γ stimulated (100 ng/ml for 24 h) THP-1 macrophage-like cells (magnification 400×). CD200R expression was clearly evident only after IFN-γ stimulation whereas LPS stimulated and unstimulated THP-1 macrophage-like cells did not revealed clear CD200R expression. **G**) CD200Hi, CD200Me and C200Lo BMMSC lines decreased significantly TNF-α secretion from THP-1 macrophage-like cells in presence of IFN-γ whereas UCBMSC lines did not. There were also significant differences between CD200Hi, CD200Me and CD200Lo BMMSC lines in their ability to inhibit TNF-α secretion of IFN-γ (100 ng/ml for 24 h) activated THP-1 macrophage-like cells whereas **H**) there were no correlation between CD200 expression and the ability of hMSCs to decrease TNF-α secretion from THP-1 macrophage-like cells in presence of LPS (100 ng/ml for 24 h). Only CD200Me 1, CD200Lo 2 and UCBMSC 2 lines had significant reduction in TNF-α secretion. Results are represented as a mean±SD of three independent replicates. TNF-α concentration in supernatants from LPS or IFN-γ activated THP-1 macrophage-like cells alone were indicated as 100% and TNF-α concentration in supernatants from co-cultures with different hMSC lines were compared to that. Before co-cultures, THP-1 macrophage-like cells were differentiated 48 hours in PMA (100 ng/ml) supplemented medium as described in materials and methods. * and ** indicates two-sample t-test p-values p<0.05 and p<0.01. !!! indicates one way analysis of variance (ANOVA) between CD200Hi, CD200Me and CD200Lo BMMSCs p-value p<0.001.

The immunofluorescent staining of THP-1 macrophage-like cells with anti-human CD200R did not show CD200R expression (**Figure 4 A,D**). In addition, the LPS activated THP-1 macrophage like cells did not reveal clear CD200R expression (**Figure 4 B,E**). However, the IFN-γ activated THP-1 macrophage-like cells showed clear expression of CD200R on cell surface (**Figure 4 C,F**). Eight hMSC lines were selected to the co-cultures with IFN-γ or LPS activated THP-1 macrophage-like cells and the TNF-α secretion was compared between co-cultures and THP-1 macrophage-like cells alone in presence of LPS or IFN-γ. In the presence of IFN-γ, CD200Hi 1 decreased TNF-α secretion from 100%±12% to 43.5±1.7% (p<0.01), CD200Hi 2 decreased TNF-α secretion form 100%±12% to 40.3.3±4.8% (p<0.01), CD200Me 1 decreased TNF-α secretion from 100%±12% to 49.3.5±1.2% (p<0.01), CD200M2 decreased from 100%±12% to 53.0%±7.8% (p<0.01), C200Lo 1 decreased TNF-α secretion from 100%±12% to 62.0%±4.4% (p<0.05), CD200Lo 2 decreased from 100%±12% to 71.9%±3.0% (p<0.05) whereas UCBMSC 1 and 2 had only minor impact on TNF-α secretion (**Figure 4G**). The ability to decrease TNF-α secretion from IFN-γ activated THP-1 macrophage-like cells differed significantly between CD200Hi, CD200Me and CD200Lo BMMSC lines (ANOVA p<0.001). On the contrary, in the presence of LPS there was no correlation between CD200 expression and the inhibition of TNF-α secretion from THP-1 macrophage-like cells (**Figure 4H**). Only few hMSC lines were able to significantly decrease TNF-α secretion from LPS activated THP-1 macrophage-like cells after 24 hour co-culture, CD200Me 1 decreased TNF-α secretion from 100%±9.0% to 79.0±5.0% (p<0.05), CD200Lo 2 decreased TNF-α secretion from 100%±78.0% (p<0.05) and UCBMSC 2 decreased TNF-α secretion from 100%±9.0 to 78.0%±7.0% (p<0.05; **Figure 4H**).

### The Interference of CD200-CD200R Axis Decreased the Capability of BMMSCs to Inhibit TNF-α Secretion from IFN-γ Activated THP-1 Macrophage-like Cells

**Figure 5:**
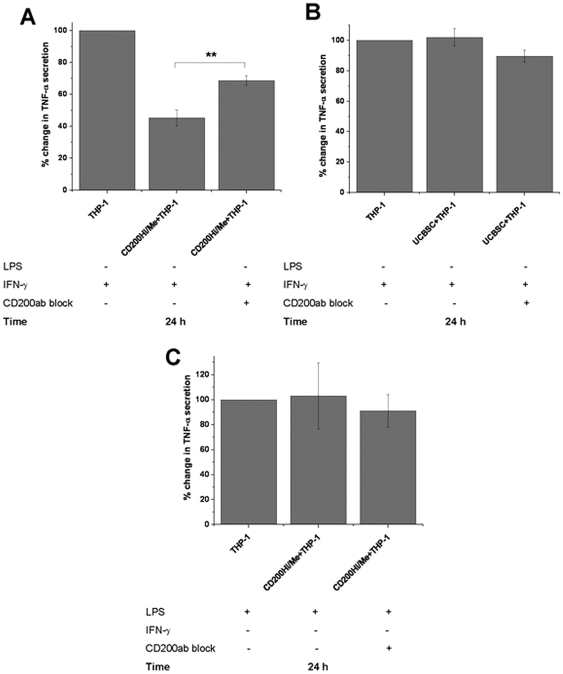
Pre-treatment of hMSCs with CD200 antibody was performed to study the role of CD200-CD200r axis in hMSC mediated immunosuppression. **A**) The ability of CD200Hi/Me BMMSCs to inhibit TNF-α secretion of IFN-γ activated THP-1 macrophage-like cells can be prevented by pre-treatment with anti-human CD200 antibody whereas the pre-treatment with anti-human CD200 antibody had no effect on **B**) UCBMSC in presence of IFN-γ and **C**) CD200Hi/Me BMMSCs when co-cultured with LPS activated THP-1 macrophage-like cells. THP-1 macrophage-like cells were activated in co-cultures with 100 ng/ml LPS or 100 ng/ml IFN-γ for 24 hours and after that supernatants were collected. Results are represented as a mean±SD of co-cultures with three different CD200Hi/Me hMSC lines and mean±SD of three independent replicates with one UCBMSC line. The blocking of hMSCs with anti-human CD200 antibody was performed by pre-incubating part of the hMSCs with CD200 antibody for 20 min at room temperature and then washed twice with PBS whereas other part of the cells remained untreated. TNF-α concentration in supernatants from LPS or IFN-γ activated THP-1 macrophage-like cells alone were indicated as 100% and TNF-α concentration in supernatants from co-cultures with different hMSC lines, anti-human CD200 pre-treated and control ones, were compared to that. Before co-cultures, THP-1 macrophage-like cells were differentiated 48 hours in PMA (100 ng/ml) supplemented medium as described in materials and methods. ** indicates two-sample t-test p-value p<0.01.

Cells from CD200Hi 1, CD200Hi 2 and CD200Me 1 lines were pre-treated with anti-human CD200 antibody and compared to untreated cells in co-culture with IFN-γ activated THP-1 macrophage-like cells. The untreated CD200Hi/Me control cells decreased TNF-α secretion from IFN-γ activated THP-1 cells from 100% to 45.3±5.0%. In the CD200Hi/Me BMMSCs, which had been pre-treated with anti-human CD200 antibody (CD200ab block), TNF-α secretion decreased from 100% to only 68.7±5.0% (**Figure 5A**). Thus pre-treatment with anti-human CD200 antibody decreased the capability of BMMSCs to inhibit TNF-α secretion when compared to untreated control cells (p<0.01). On the contrary, pre-treatment of cells from UCBMSC 1 line with anti-human CD200 antibody did not have a significant impact in the ability to decrease TNF-α secretion from IFN-γ activated THP-1 macrophage-like cells when compared to untreated ones (**Figure 5B**). The TNF-α secretion (mean±SD of three independent replicates) changed from 100% to 102±5.7% in co-culture with untreated control cells and from 100% to 89.7±4.0% in co-culture with UCBMSCs which had been pre-treated with anti-human CD200 antibody (**Figure 5B**). In addition, there were no significant differences in the ability to decrease TNF-α secretion from LPS activated THP-1 cells between untreated CD200Hi/Me and CD200Hi/Me which had been pre-treated with anti-human CD200 antibody. TNF-α secretion changed from 100% to 103±26.5% in co-culture with un-treated controls and from 100% to 91.0±12.0% in co-culture with anti-human CD200 antibody pre-treated CD200Hi/Me BMMSCs respectively (**Figure 5C**). These results also indicate that IFN-γ primed THP-1 cells responded to BMMSCs in a CD200-CD200R dependent manner.

## Discussion

The immunomodulative properties of hMSCs from different sources have been widely studied in several immune cell models [11]–[13], [15], [21], [22]. However, the interaction between hMSCs and human macrophages has received little attention even though macrophages are key players in innate immunity [37], [38]. Studies on murine cells are also in line with findings in human cells [42]. The present study has, to our knowledge, been the first to ever to demonstrate the variable CD200 expression from 20 different hMSC lines. Moreover, we have also been able to elucidate the role of CD200-CD200R axis in hMSCs mediated immunosuppression of THP-1 macrophage-like cells. THP-1 monocytes can be differentiated into macrophage-like cells by various agents, such as PMA and vitamin D, and these cells have shown to possess properties similar to tissue resident macrophages [43]–[46]. In addition, others have previously shown that THP-1 macrophage-like cells can be used to study the interactions between macrophages and hMSCs [37].

The expression of CD200 in hMSCs has been previously published [27], however we have been able to show also differences in the number of CD200 positive cells in samples cultured from different donors. In addition, our study revealed that none of the three UCBMSC lines expressed CD200. However, all lines used in this work showed characteristics cell surface antigens for hMSCs. Moreover, the extensive passaging of the CD200 positive hMSCs did not have any effect on CD200 expression with reference to the characteristics of hMSCs. No systematic correlation between immunosuppression efficiency and age of BMMSC donors was evident, which could, at least partly, be due to insufficient sample size. However, the CD200 expression was shown to correlate with the ability of BMMSCs to inhibit TNF-α secretion of THP-1 macrophage-like cells in presence of IFN-γ even at rather low amount of samples which could underline the physiological significance of such signaling. The CD200 could be a potential marker of a more immunosuppressive hMSC population.

The reason for the differences in CD200 expression between UCBMSCs and BMMSCs and also between BMMSC lines remains to be elucidated. One explanation for the lack of CD200 in UCBMSC may be the differences in the micro environment *in vivo*, since BMMSCs reside in close proximity with macrophage progenitors unlike UCBMSCs [47], [48]. This may also explain the relatively low capability of UCBMSCs to inhibit TNF-α secretion from THP-1 macrophage-like cells when compared to BMMSCs. Moreover, CD200-CD200R axis also participates in the regulation of bone mass, which may be one plausible explanation for the differences in CD200 expression between UCBMSCs and BMMSCs [49].

Interestingly, the ability of hMSCs to suppress TNF-α secretion of IFN-γ stimulated THP-1 macrophage-like cells was significantly greater when compared to LPS activated ones. IFN-γ did not reveal clear induction of TNF-α secretion from THP-1 macrophage-like cells unlike that of LPS. This observation is in line with a recent study where proteome bioprofiling of differently activated macrophages were studied [10]. The same study also showed that IFN-γ and LPS caused different protein profiles which were distinguished from basal macrophages [10]. These results clearly demonstrate the differences in the activity of macrophages between IFN-γ and LPS stimulation, in which the IFN-γ seems to prime macrophages whereas LPS causes more drastic activation. Even thought IFN-γ did not cause a clear increase in TNF-α secretion from THP-1 macrophages, the key role of IFN-γ in many inflammatory conditions is well known. For instance, IFN-γ together with LPS is prerequisite for the development of inflammatory bowel disease [8] and the role of IFN-γ has also been demonstrated in development of atherosclerosis [6], [9] and in Crohn's disease [7], which are only a few of the inflammatory conditions in which macrophages are also implicated.

In previous studies, LPS has been used as an activator for macrophages and it has been shown that hMSCs inhibit TNF-α secretion [37], [38]. Evidently, our results are not in line with such data, possibly due to the differences in co-culture protocols. Previous studies have measured TNF-α production after 4 h LPS stimulation whereas we used a 24 h time point. More specifically, these observations may suggest that the ability of hMSCs to inhibit LPS stimulated macrophages may be limited only to the early phase whereas in presence of IFN-γ hMSCs have more prolonged effect on TNF-α secretion from macrophage-like cells. It is also noteworthy, that many pro-inflammatory factors, such as TNF-α, IFN-γ and LPS modulate the immunosuppressive properties of hMSCs [20], [21], [50]. Accordingly, it is also possible that LPS and IFN-γ may differently modulate the immunosuppression potential of hMSCs, which may explain the differences. IFN-γ has, for example, been shown to regulate mesenchymal stem cells via activation of indoleamine 2,3 dioxygenase, which suggests that a complex network of inflammation control could exists *in vivo* [21], [51]. However, such a hypothesis is extremely challenging to prove in our study, as during co-culture hMSCs are exposed to several different cytokines which most likely have combinatorial effects. Regardless, our observations suggest that also other activators than LPS alone, should be considered, when the immunosuppressive potential of hMSC-based cellular products is determined. Supporting such a conclusion, the previous study has also shown that using combination of IFN-γ and LPS leads to a more differential activation of macrophages and the presence of both stimulators are needed to develop bowel inflammatory disease [8].

THP-1 macrophage-like cells expressed CD200R only after PMA treatment following IFN-γ activation. This observation is in accord with the findings which showed that CD200Hi BMMSC lines decreased the TNF-α secretion from THP-1 macrophage-like cells in presence of IFN-γ more efficiently when compared to CD200Lo BMMSC lines. However, there were no differences when these cell lines were co-cultured in the presence of LPS. Also these findings are well in line with previous data which has demonstrated that the CD200R agonist had immunosuppressive effect only when mouse peritoneal macrophages were activated with IFN-γ, whereas LPS-stimulated ones were unaffected [52]. However, although CD200 negative UCBMSCs showed the weakest inhibition capacity of TNF-α secretion, the present study can not conclude that the lack of CD200 can explain the differences as the UCBMSCs represent a different hMSC type. Conflicting results have also been published on the role of the CD200-CD200R axis in THP-1 cells [53], [54]. The lack of CD200R expression in the study by Salata et al. [54] may possibly be due to different THP-1 differentiation and activation protocols, as we have used longer PMA treatments with higher concentration in accordance with Langlais et al [53]. In addition, a recent study has shown that the maturity of THP-1 macrophages is related to the duration of PMA treatment and the duration of the incubation time without PMA [55].

The present study has shown that the interference of CD200 binding to CD200R weakened significantly the ability of BMMSCs to inhibit TNF-α secretion from IFN-γ activated THP-1 macrophage-like cells. On the other hand, the interference of CD200-CD200R interaction did not fully prevent the capability of hMSCs to suppress TNF-α secretion of IFN-γ activated THP-1 macrophage-like cells but decreased this capability to the same level as was evident in CD200Lo BMMSC lines. This suggests that BMMSCs may also have other mechanisms for altering TNF-α secretion of THP-1 macrophage-like cells in addition to the CD200-CD200r axis. The anti-human CD200 antibody used in the present study was not specifically produced for blocking experiments, but our results showed that the anti-human CD200 antibody pre-treatment had no effect on the capability of CD200Hi/Me BMMSC to inhibit TNF-α secretion in presence of LPS (CD200R negative THP-1 macrophage-like cells) or UCBMSCs to inhibit TNF-α secretion in presence of IFN-γ (CD200 negative), which support the reliability of our results.

The CD200-CD200r axis represents one specific interaction between hMSCs and immune cells in the wide and complex set of different immunosuppressive mechanisms which interact parallel. The exact role of the CD200 in that process remains to be elucidated and needs to be clarified *in vivo* inflammatory disease models in future. It is also possible, that CD200 may just be a sign of a cell population with distinct immunosuppressive capacity. It is to be noted, that this capacity can not be detected by the conventional hMSC characterization panel. By using different immune cell models, future studies should also focus on testing the immunosuppressive potential of CD200Hi, CD200Me, CD200Lo and UCBMSC, as previous studies have suggested that CD200-CD200R axis may not be as relevant in modulation of adaptive immunity [56], [57]. In the present study, all hMSC lines did induce significant IL-10 secretion from THP-1 macrophage-like cells, which was in line with previous studies [37], [38], but it was not in line with CD200 expression (data not shown). Hence, as the immunological mechanisms are very complex there is still much room for other, CD200 independent immunosuppressive mechanism for hMSCs. Moreover, further characterization of CD200 positive BMMSCs in future is critical because CD200 has also been linked to cancer stem cells and tumors [58], [59].

To conclude, this study has clearly clarified for the first time the role of CD200 in BMMSCs mediated inhibition of TNF-α secretion from macrophages in the presence of IFN-γ. CD200Hi BMMSCs showed higher inhibition of TNF-α secretion from THP-1 macrophage-like cells when compared to CD200Lo BMMSC. The results indicate that CD200 could be a potential candidate for predicting the immunosuppressive activity of cultured BMMSCs in clinical conditions, where activated macrophages play a key role.
